# Supplementary material for: Are carbon emissions trading and green financial instruments synergistic? -Comprehensive quantitative research based on content analysis
Source: PLoS One. 2024 Mar 7;19(3):e0298601. doi: 10.1371/journal.pone.0298601 (PMC10919601; doi:10.1371/journal.pone.0298601)
Supplement: S1 Data — (ZIP) [file pone.0298601.s001.zip › Data/地级市Z_F工作报告.zip!/╔┬╬≈/═¡┤¿╩╨/└┤╫╘╣1⁄2╓┌║┼í╛┬φ┐╦╩2╛▌═°í┐_2012.docx]

各位代表：

　　现在，我代表市人民政府向大会作工作报告，请予审议，并请市政协委员和其他列席人员提出意见。

　　一、十五届人大一次会议确定的目标任务执行情况

　　过去的一年，在省委、省政府和市委的正确领导下，我们紧紧围绕提速发展、富民强市的主题，紧扣加快转变经济发展方式、推进资源型城市转型的主线，按照“创特色、比速度、争一流”的总体要求，积极应对经济环境复杂变化，加大解决突出问题力度，圆满完成了市十五届人大一次会议确定的各项任务，顺利实现了“十二五”开局之年高点起步的目标。

　　全市经济持续快速增长，主要经济指标增速保持第九年位居全省前列。全年实现生产总值234.53亿元，增长16%，增速位居全省第二位；财政总收入38.36亿元，增长35.7%，增速位居全省第四位；地方财政收入18.89亿元，增长37.4%，增速位居全省第三位；全社会固定资产投资完成145.96亿元，增长32.6%，增速位居全省第二位；城镇居民人均可支配收入18775元、增长18.2%，增速位居全省第三位，农民人均纯收入6077元、增长26.9%，增速位居全省第五位，分别高出全省平均水平537元和1048元。

　　——项目建设成效显著，发展后劲不断增强。坚持把项目建设作为提速转型发展的总抓手，努力破解项目建设的瓶颈制约。全年实施的80个重点项目，新开工项目22个、续建项目37个，完成投资66亿元，20个项目建成投产或投运。民间投资日趋活跃，投资额达到68.53亿元，增长59.5%。延西高速西安至铜川段、陕煤建司管件厂迁厂扩能一期、矿山120万吨水泥粉磨站、白石崖煤矿45万吨技改、110千伏柳湾智能变等项目已建成投产或投运；声威第三条日产4500吨水泥熟料生产线、耀旬二级公路改建、照金香山景区建设、铜川矿务局棚户区改造等项目达到年度计划进度；30万吨碳素、陕汽零部件加工基地二期、大唐养生园、国家矿山救援铜川基地等项目已开工建设。华能电厂二期、董家河循环经济产业园、国电热电联产、油气资源开发等重大项目前期工作取得新进展。

　　——工业经济较快增长，运行质量明显提高。我们把保工业增长作为工作重点，强化工业运行协调，全力克服诸多因素对工业经济的严重冲击，工业经济保持平稳较快发展。全市规模以上工业实现增加值123.81亿元，增长19%。在统计的25种主要工业产品中，有20种产品产量保持增长，其中原煤产量2363万吨、增长10.8%，水泥1429万吨、增长45.8%，发电63.18亿千瓦时、增长6.3%。电解铝在市场和电价双重冲击下仍保持生产稳定，产量达到23.35万吨。工业产销率97.7%。全市规模以上工业企业实现主营业务收入313.8亿元，增长35.1%；实现税金17.44亿元，增长13.5%。

　　——“三农”工作进一步加强，特色农业不断壮大。坚持把“三农”工作作为重中之重，认真落实各项强农惠农政策。各级财政支农资金6.93亿元，增长25.7%。全市耕地保有量148万亩，粮食连续八年丰收，总产达到21.44万吨。新栽鲜干果经济林14万亩，总面积达到206.12万亩，其中水果面积88.19万亩，总产62.95万吨，优果率82%。肉、蛋、奶产量分别达到1.55万吨、1.58万吨和2.55万吨，分别增长5.6%、14.5%和8.1%，畜禽规模化饲养率达70%。新启动20个新农村示范村和20个扶贫开发重点村建设，1.89万人实现脱贫。龙潭水库导流泄洪洞工程完工；5.5万农村群众的安全饮水问题得以解决。新修通村通组产业路63公里，新建户用沼气池2400口，移民搬迁5175人，农村危房改造4230户。农村居民进城落户2.04万人。我市特色农业优势不断显现，中省主流媒体对我市苹果、大樱桃、奶牛等特色产业和新农村建设进行了专题报道。

　　——成功举办中医药文化节，现代服务业快速发展。在中省有关部门及社会各界的大力支持下，通过全市上下的共同努力，首届中国孙思邈中医药文化节取得圆满成功，展示了铜川深厚的文化底蕴，为养生保健产业发展搭建了良好平台，扩大了对外知名度和影响力，树立了铜川的良好形象，在省内外产生了广泛的影响。围绕办好文化节，狠抓“二园四区四基地”建设，强力推进旅游、餐饮、商贸等服务业发展，制定出台了加快现代中医药产业发展的决定、商贸物流发展专项规划和加快新区商贸服务业发展实施意见，大唐养生园累计完成投资3亿元，照金香山景区累计完成投资5.26亿元，药王大道建成通车，药王山被评为国家4A级旅游景区，被中华中医药学会确定为“孙思邈学术医德养生思想传承基地”。全市文化产业实现增加值3.27亿元，增长33.4%，增速位居全省第二位。全年接待国内外游客560万人次，实现旅游综合收入19.11亿元，分别增长13%和82%。全社会消费品零售总额53.26亿元，增长17.1%。

　　——“四城联创”深入推进，城市品位不断提升。投资1.35亿元，实施创卫项目22类367个。农村户厕改造4.5万户，在全省率先实现了以市为单位的农村户改厕全覆盖。持续开展了市容环境专项整治、210国道沿线综合整治，拆除了一批有碍观瞻破旧建筑，顺利通过了创国卫省级考核鉴定。加强林业重点工程建设和区域绿化工作，植树造林20.07万亩，治理水土流失161.32平方公里，森林覆盖率达到44.8%，完成了创建全国绿化模范城市阶段性任务。实施了“千绿万红百增艳”城市绿化工程，新增城市绿地100万平方米，建成区绿化覆盖率42.5%、绿地率36.5%，人均公园绿地面积10.26平方米，我市成功创建为省级园林城市。省级环保模范城市创建工作扎实推进，单位GDP能耗预计下降3.6%，降幅位居全省前列；二氧化硫、化学需氧量和氨氮排放量分别削减2.19%、1.95%和2.1%。北市区污水处理厂升级改造主体工程完工，新耀中水回用工程已正式投运，城市污水处理率80%、垃圾处理率87%。新发展天然气用户11307户，城市气化率73%，新建清洁能源锅炉32台，为1万户困难群众发放了煤票补贴，城市空气质量二级以上天数达到328天。

　　——改革开放不断深化，发展活力持续增强。启动了市物资开发公司等3户国有企业民营化改制工作。开展了涉农无形资产质押贷款业务。医药卫生体制改革不断深化，国家基本药物制度全面实施，完成了公立医院改革和县镇卫生一体化管理改革试点工作。不断深化国库集中支付改革，在9个市级部门推行了预算公开试点，在10个市级预算单位启动了公务卡制度改革试点。完成了工商局、质量技术监督局移交管理工作。认真落实鼓励全民创业的各项政策，新增个体工商户3157户、私营企业438户，非公有制经济占比达46.2%。加强对外交流与合作，坚持走出去、请进来，与24个省上厅局和单位签订了战略合作协议，与华能、国电、旺旺、中石化、泛华等大企业、大集团的合作不断深入，赴天津、河北、江苏、广东等地开展了专题招商，组团参加了西洽会、农高会、厦洽会等展会招商活动。全年签订招商引资项目145个，涉及资金528亿元，115个项目进入实施阶段，累计到位资金91.7亿元。实现外贸进出口总额1016.8万美元，增长107.5%，其中出口836.3万美元，增长114%，增速位居全省第一位。

　　——倾力推进民生工程，人民群众得到更多实惠。我们在财力有限的情况下，坚持把保障和改善民生作为重中之重，能早安排则早安排，能高标准则高标准，一些民生工作走在了全省前列。全年新增财力85.4%用于民生，投入23.15亿元，实施民生工程项目440个，已建成333个。在全省率先实现城乡居民社会养老保险一体化全覆盖，城乡居民缴费标准、财政补贴、保险待遇、基金管理、经办服务“五统一”，参保人数已达29.09万人，5.85万人领取了养老金。将全市国有和部分集体企业2902名“老工伤”人员纳入了统筹管理，解决了21211名未参保集体企业超龄人员养老保险补贴问题。城镇基本医疗保险参保36.8万人，城镇企业职工基本养老保险扩面3013人，新增城镇就业1.67万人，城镇登记失业率3.78%，均超额完成省下达的目标任务。在全省率先实现了市、区县、乡镇、社区四级社会保险机构和网络全覆盖。全市新开工保障性住房181.6万平方米，竣工56万平方米，新增廉租住房租赁补贴2504户，保障住房困难家庭9280户，被省委、省政府评为2011年度全省保障性住房建设一等奖，获得奖金5000万元；王益区被评为“十佳县”，获得奖金600万元。完成20个城市社区服务体系项目和60个农村社区服务中心建设，4个区县级中心敬老院全部建成。城乡低保做到应保尽保，保障困难群众11.13万人。城乡优抚对象待遇全面落实。争取救灾资金4165万元，5.2万受灾群众的基本生活得到妥善安排。王益区创建省级教育强区通过省政府评估验收，实现了教育强区零的突破。完成了30所农村中小学生活设施改造、52所中小学校舍安全工程年度建设任务，学前一年免费教育全面实施，建成了王益区城市幼儿园和8所公办乡镇中心幼儿园。资助家庭经济困难高中生13373人次、中职生3814人次、新录取家庭困难大学生400人。我市被评为全国科技进步考核合格市，获得全省3项科技进步奖，全年开发转化科技成果24项。新型农村合作医疗参合率96.99%，财政补助标准由每人每年120元提高到200元。新建农家书屋150家。实施了20个行政村农民体育健身工程和10个社区体育器材配送工程，成功举办了市第四届老年人运动会、第五届农民运动会。出台了一系列稳定物价的措施，向低收入群众及特困群体发放价格补贴2581万元，居民消费价格指数稳定在104.9%。

　　——推进社会管理创新，社会大局和谐稳定。完善应对突发事件机制和重大事项社会稳定风险评估机制，最大限度地增加和谐因素。启动实施了“六五”普法工作，开展了第八次村委会换届选举，完成了撤乡并镇工作。妥善处置“10·16”田玉煤矿重大事故，采取更加严格的措施，狠抓煤矿安全生产和道路交通等重点领域安全专项治理，强化校园安全特别是校车管理，加强各类安全隐患排查整改，努力稳定全市安全生产形势。食品药品监管工作不断加强。深入推进“平安铜川”建设，国防动员能力和民兵预备役建设水平不断提高，维稳处突能力有效提升，严打整治斗争成效显著，“清网行动”战绩位列全省第二，群众安全感明显增强，在全省群众满意度调查中，群众满意率居全省第四，“两代表一委员”满意率居全省第一。深入开展领导干部接访、矛盾纠纷排查、信访积案化解等活动，完成了“一控制、两下降”目标，在全省设区市中唯一实现“三无”区县全覆盖。

　　——政府自身建设不断加强，服务水平进一步提高。完善了《政府工作规则》，健全了科学决策、民主决策机制。坚持依法行政，自觉接受人大及其常委会的监督和政协的民主监督，全年办理人大代表建议60件、政协提案174件，办复率、回访率、满意率均达到100%。积极推进政务公开和电子政务，全年办理回复网民留言509件，一些涉及群众切身利益的热点难点问题得到较好解决。大力开展“为民服务零距离”活动，强化责任和工作落实，机关作风不断改进。进一步规范政府采购工作，严格控制楼堂馆所建设和“三公”经费，清理整顿公务用车，廉政勤政建设得到进一步加强。

　　各位代表，过去的一年，我市其他方面的工作也取得了明显进步。全市上下呈现出经济提速发展、社会和谐稳定、干部心齐劲足、人民幸福安康的可喜局面。这些成绩的取得，是市委统揽全局、科学决策的结果，是市人大、市政协和社会各界监督支持的结果，更是全市上下同心同德、奋力拼搏的结果。在此，我代表市人民政府，向全市广大干部群众、驻铜部队指战员、武警官兵、公安干警，向各民主党派、各人民团体、社会各界人士表示崇高的敬意和衷心的感谢！

　　在充分肯定成绩的同时，我们也清醒地认识到，我市经济社会发展仍然面临不少困难和问题，主要是：经济结构不尽合理，非公有制经济占比低，县域经济实力不强，服务业发展滞后，经济外向度偏低。经济发展方式比较粗放，资源性产业比重大，转型发展任务艰巨；受市场、政策等因素影响，水泥、铝业等传统优势产业发展受困，保增长的压力增大。涉及群众利益的住房、就业、教育、医疗、交通、安全等诸多问题还需下更大气力解决。干部作风有待改进，政府效能建设、勤政廉政建设需要进一步加强。对此，我们将采取更加有力的措施，切实加以解决。

　　二、2012年的主要目标和工作任务

　　当前，我市正处在加快转型发展的重要战略机遇期，国家和省委、省政府对铜川转型发展寄予厚望。市十一次党代会描绘了未来五年的发展蓝图，提速发展、加快转型、建设休闲养生城市的思路更加清晰，干部群众求富思进的热情更加高涨。我们一定要抢抓机遇，乘势而上，顺势而为，坚持加快发展第一要务不动摇，坚持维护稳定第一责任不松懈，坚持保障和改善民生力度不减弱，稳中求进，好中求快，努力推动经济社会发展迈上新台阶。

　　做好今年的政府工作，要以科学发展观为指导，全面贯彻党的十七大以来的重大部署及省委十一届八次全会、市十一次党代会精神，以“提速发展、加快转型、富民强市”为主线，大力实施工业强市、文化兴市、生态立市三大战略，全力抓好“科学发展双十行动”，扎实推进能源、果业、现代建材、装备制造、食品加工、休闲养生六大产业基地建设，创特色、比速度、争一流，加快转变发展方式，深化改革开放，统筹城乡发展，大力保障和改善民生，加快推进经济强、文化兴、生态美的全国知名休闲养生城市建设。

　　全市经济社会发展的主要预期目标是：生产总值达到276亿元，增长14.5%；全社会固定资产投资达到187亿元，增长28%；财政总收入达到41.8亿元，地方财政收入达到18.87亿元，同口径均增长18%；城镇居民人均可支配收入达到21780元，增长16%；农民人均纯收入达到7170元，增长18%；居民消费价格指数控制在104.8%以内，城镇登记失业率控制在4.5%以内，人口自然增长率控制在5.5‰以内，万元生产总值能耗下降3.5%，主要污染物排放指标控制在省上规定的范围内。

　　（一）确保固定资产投资快速增长。

　　项目带动、投资拉动仍是我市今后较长时期内必须坚持的工作战略。扎实开展“项目落实年”活动，进一步加大项目建设力度，在重大项目建设上实现新突破。

　　突出抓好重点项目建设。举全市之力，大力实施“科学发展双十行动”，加快重点项目建设进度。今年安排市级重点项目115个，22个项目建成投产或投运。市级重点项目总投资988.65亿元，今年计划完成投资129.2亿元，其中“科学发展双十行动”总投资593.87亿元，今年计划完成投资71.44亿元，确保54个重点项目开工建设、49个续建项目达到计划进度。加快12个前期项目规划、环评、土地报批等工作，争取年内开工建设。

　　破解项目建设瓶颈制约。把解决项目用地作为重点，坚持集约节约用地，加快项目用地报批、供地速度，加大对闲置土地的清理力度，积极实施城乡建设用地增减挂钩。充分发挥市开发投资公司等融资平台的作用，加强政银企合作，加大金融对项目建设的支持力度。发挥财政资金引导作用，鼓励社会资本投资重点项目建设。全力做好项目环境保障工作，树立项目建设“一盘棋”的思想，该配套的基础设施尽快配套到位，该落实的优惠政策坚决落实到位，为项目建设营造良好的环境。

　　加大项目争取力度。瞄准中省投资方向和战略重点，结合我市资源型城市转型，精心策划、包装、储备一批带动力强的大项目、好项目。市财政安排项目前期费用和奖励资金2200万元，加大对项目争取、项目建设的考核和奖励。抓好中省支持资源型城市转型各项政策的落实工作，使更多项目挤进中省盘子，确保争取中省政策性资金10亿元以上。加强与省级有关部门的衔接，认真抓好省促进铜川资源型城市转型发展领导小组第二次会议纪要和各项战略合作协议的落实工作，确保既定项目落到实处。

　　（二）确保工业经济快速增长。

　　工业是我市的经济支柱，工业强则经济强。要继续保持全市经济持续快速增长，实现主要经济指标增幅位居全省前列的“第十年”，就必须把保工业增长放在更加突出的位置，坚定不移实施工业强市战略。市财政安排7700万元，用于保产业、促项目、增效益，使全市规模以上工业增加值增长18%以上。

　　千方百计保增长。加强工业运行监测和市场预警，继续坚持“旬报告、月分析、季考核”制度，加强对重点行业和企业的协调服务，做好生产要素保障工作，确保骨干企业开足马力生产。在确保安全生产的前提下，加快煤炭资源整合，对验收达标的矿井有序恢复生产。研究应对水泥市场疲软的政策措施，积极开拓市场，努力提高市场占有率。启动国家水泥产品质量监督检验中心建设。抓好节能降耗，延伸铝业产业链，有效应对电价对铝业的冲击。继续做好电煤保障工作，确保华能铜川电厂满负荷运转。加强企业内部管理，加大技术改造力度，积极培育骨干企业，力争新增规模以上工业企业10户以上。

　　加快工业项目建设。加强与华能、国电、陕煤化、陕有色的合作，加快煤电铝联产联营项目建设，推进煤炭就地转化、铝产业延伸和水泥、陶瓷产业升级。开工建设耀西煤矿、冶平煤矿、国电热电联产和30万吨电解铝项目，加快华能电厂二期前期工作。开工建设30万吨铝合金项目，争取建成30万吨碳素项目。确保秦岭第二条日产4500吨水泥熟料生产线和华原100万吨水泥粉磨站建成投产，康元日产4500吨特种水泥熟料生产线开工建设。现代工艺陶瓷、大鹏陶瓷生产线建成投产。积极培育接续产业，陕煤化PVC管材、大秦铝合金生产线、明鑫耐磨材料生产线建成投产，加快陕汽零部件加工基地二期工程建设，开工建设陕煤建司管件厂迁厂扩能二期、旺旺三期、米尼照明产业园等项目。加快推进宜君油气资源开发、印台油页岩资源开发和阿庄风电场建设。积极推进第四座330千伏变电站和电动汽车充换电站建设，完成35千伏照金变、瑶曲变增容改造工程。

　　提高工业园区承载力。完善园区管理机制，强化各区县抓工业保增长的意识和发展园区经济的意识，加大工业园区工业增长速度、工业总产值指标的考核排序奖励力度。市财政安排4000万元，支持各区县用于工业园区发展，引导金融资金、社会资本参与园区建设，完善园区基础设施。加快园区产业体系建设，加大招商引资力度，狠抓大项目、大企业的落地，力争2―3个有规模的产业类项目落地建设，使园区真正成为带动县域经济发展的增长极。

　　（三）全面加快现代农业发展步伐。

　　认真贯彻中央一号文件精神，加强科技创新，坚持“三化同步”，始终不渝地推动各项强农惠农富农政策的落实，千方百计增加农民收入。市县财政支农资金达到3.23亿元，较上年增长29.9%，确保实现农业增产、农民增收、农村繁荣。

　　大力发展现代特色农业。实施粮食单产提升和高产创建工程，粮食总产稳定在19万吨以上。扎实抓好果业提质增效工程，全年新栽水果8万亩、干杂果4万亩，更新改造果园8万亩，新建3个万亩现代果业示范区，辐射建设20个千亩现代果业示范区，苹果优果率83%以上。坚持绿色增长和生态富民相结合，壮大林业产业，发展林下经济。大力推进奶牛、肉牛、生猪的规模化、标准化养殖，创建5个省部级标准化规模养殖示范场。实施好菜篮子工程，稳定蔬菜市场供应。

　　加快现代农业园区建设。启动实施“现代农业园区建设年”，出台扶持农业园区发展的政策措施，每个区县要快节奏推进、高标准建设2个以上特色鲜明、效益突出的市级现代农业园区。继续支持周陵现代农业科技示范园建设，建成2000亩大樱桃基地。启动建设印台乡苹果、棋盘镇核桃等观光农业示范乡镇和马咀设施樱桃观光园、陈坪樱桃观光园及孟家塬鲜桃观光园。鼓励引导农户以转包、租赁、入股等方式流转土地承包经营权。加快培育农业产业化龙头企业和农民专业合作社等市场主体，实施龙头企业和合作社“双十强”工程，加强新型农民培养，聘请首席农业专家，加快农业科技成果推广应用，提高农民组织化、专业化程度和农业产业化水平，创建国家级农业龙头企业1个、省级农业龙头企业3个、市级示范合作社15个。建设20个乡镇（区域）农产品质量安全检测站，在所有乡镇设立农产品质量安全监管站。

　　改善农村生产生活条件。加大强农惠农富农工作力度，确保各项补贴按时足额发放到位。加快龙潭水库枢纽工程建设和库区移民工作。完成枣园、沟西等6座小型水库除险加固工程建设任务。加强耕地保护，新修改造基本农田、新增节水灌溉面积各2万亩，完成农业综合开发土地治理2.2万亩。加强扶贫开发工作，完成照金革命老区连片开发项目建设。继续抓好农村安全饮水工程建设，解决3万农村群众安全饮水问题。建立健全管理机制，强化农村饮水工程管理和维护工作。建设县乡公路40.2公里、通村公路60公里、村庄道路50公里，农村危旧房改造4000户。加快农村电网改造升级工程实施步伐。

　　（四）强力推进第三产业发展。

　　第三产业是我市经济发展的短板，我们要加大工作力度，加大财政投入，加大政策支持，多措并举，促进第三产业快速发展。力争使服务业增加值增长14.5%以上。

　　大力发展休闲养生产业。加快“二园四区四基地”建设，市财政安排5000万元用于中医药产业发展，安排1000万元用于旅游产业发展。按照建设全国知名养生主题创意园区的目标，加快大唐养生园建设。规划建设中医药产业园，引进一批休闲养生项目，推进中医药养生保健产业集群化、园区化发展。完成药王山大景区规划编制工作，建成药王广场，加快药王故里建设，加大实施以中药材树种为主的景区绿化工程，新建一批休闲养生设施，启动实施景区规划范围内水泥厂、居民搬迁工程。加强与陕文投、陕煤化集团的合作，加快照金景区建设。完善玉华宫景区服务功能，提升景区品位。加快陈炉特色旅游古镇建设。大力推进中药材铜川种植、流通、研发、检测四大基地建设，新增中药材种植面积1万亩，加快药王方剂开发和药品、保健品研发，建设方舟药用种植研发生产基地，开工建设中国中药材铜川检测基地和孙思邈中医研究院。加强与中省有关部门的协作，精心做好第二届中国孙思邈中医药文化节筹备工作，搞好活动策划，加强宣传推介，加快活动场馆等硬件设施建设，力争再办出一届有创意、有特色、高水平的节庆活动。

　　加快商贸物流业发展。认真实施全市商贸物流发展专项规划和加快新区商贸服务业发展的实施意见，市财政安排1000万元支持商贸物流发展。开工建设西北耐物流中心，积极推进西北现代果业物流园区项目，加快建设二手车交易市场。开工建设3个五星级宾馆、3个快捷酒店和5个商场、2个写字楼，确保深圳美佳华（沃尔玛）开工建设。建成后新增床位1870张、商业面积13.17万平方米、办公面积10.4万平方米。新区金谟西路步行街建成营业，并举办红酒节活动。建成有特色、标准化的农家乐60户，满足群众多元化需求。抓好农民建房、农用汽车和农机具下乡等工作，促进农村消费。加快发展物业服务、社区服务、家政服务和养老服务等面向民生的第三产业，培育新的消费热点。确保社会消费品零售总额增长20%以上。

　　（五）大力保障和改善民生。

　　坚持以人为本、以民为先，构建以民生事业为基点的公共财政框架，新增财力80%以上用于民生，从群众最紧迫、最需要、最关心的问题入手，以公共服务均等化和城乡一体为方向，尽最大努力办好顺民意、解民忧、惠民生的实事，让城乡群众共享改革发展成果！

　　倾力抓好民生十大工程。一是安居工程。投资4.36亿元，开工建设保障性住房1.5万套、110万平方米，竣工1万套，安置住房困难家庭1万户。新增住房租赁补贴3000户。城市避险搬迁3000户。完成地质灾害点群众搬迁551户2193人。二是医疗卫生工程。投资1.37亿元，开工建设市新区医院二期工程、市中医医院新区项目、美国ICT肿瘤医院项目，建成精神卫生康复中心。建成后新增病床1700张。南北市区新建18座标准化公厕。三是教育惠民工程。投资1.11亿元，完成7所城市公办幼儿园和19所乡镇中心幼儿园建设任务；资助400名新录取的家庭经济困难大学生，为6000名家庭经济困难大学生办理生源地助学贷款；加强校车安全管理，全市校车统一标识，筹措2000万元资金购置专用校车。解决农村家校3公里以外中小学生午餐问题。四是公共文化设施工程。投资2亿元，开工建设体育中心、文化艺术中心（大剧院），为市民提供游泳、篮球、网球、羽毛球、乒乓球和影视娱乐等文化体育健身服务。开工建设城市规划展览馆。五是增绿碧水工程。投资1.65亿元，开工建设阳光广场至朝阳公园带状休闲广场、川口山体游园、印台区济阳公园、宜君县龙山公园，完成新区牡丹园扩建一期工程，新增绿地60万平方米。完成南市区碧水工程规划和新区景观设计，实施沮河景观、赵氏河湿地公园景观工程，开工建设新区城市中心公园药王湖、大唐养生园生态湖、药王山景区生态湖等工程，建成后形成水面20余万平方米。六是交通畅通工程。投资28.48亿元，开通西铜公交线路，开工建设合凤高速新区至照金段、延西高速铜黄段，210国道改线先行开工建设黄堡段，耀旬二级公路全线建成投运。新增出租车60辆，新增公交车25辆，新开公交线路2条，线路里程48公里。新增停车位1200个。七是扶贫搬迁工程。投资1.75亿元，启动实施20个整村推进建设项目；完成贫困人口移民搬迁5000人；启动实施新区2­－3个城中村改造项目，开工建设村民住宅5万平方米。八是流通网络工程。投资3100万元，建设1个蔬菜配送中心，配备10辆蔬菜流动直销车，建设25个社区蔬菜直营店、4个农贸批发市场、5个社区标准化菜市场，建设80个放心早餐流动销售网点，新增营业摊位800个，提供就业岗位1500个。九是便民服务工程。投资3000万元，建设市、区县便民服务大厅、乡镇（街道）便民服务中心、行政村（社区）便民服务站，受理与群众利益密切相关事项，四级联动，一厅式办公、一条龙服务。十是平安建设工程。投资4800万元，强化治安防控体系建设，新增2624个视频监控探头，公共场所覆盖率达到80%。加强群防群治队伍建设，加大街面、住宅小区、人员密集场所巡逻频度，有效遏制盗窃、抢劫等犯罪，提升群众的安全感。

　　各位代表，就业是民生之本，我们要加大对困难群体的就业援助，做好高校毕业生、复员退伍军人、进城落户农村居民的就业工作，多渠道增加就业岗位，努力推进城乡充分就业。全年新增城镇就业1.6万人，下岗失业人员再就业5000人，发放小额担保贷款2亿元。切实保障劳动者合法权益，推进各类企业加快建立工资集体协商制度，落实新的企业最低工资标准，健全职工工资正常增长机制和支付保障机制，提高企业退休人员养老金标准。解决好社区和村组工作人员的待遇问题。确保城乡困难群众和特殊群体各项补助按时足额发放。抓好城乡居民房屋确权工作，切实增加群众财产性收入和经营性收入。继续扩大社会保险覆盖面，着重解决集体企业职工养老遗留问题，城乡居民社会养老保险参保率达到97%以上，城镇基本医疗保险参保人数稳定在36.8万人，煤矿、建筑等高危行业职工全部纳入工伤保险范围。将新型农村合作医疗和城镇居民基本医疗保险财政补助标准提高到250元，并同步提高报销比例，扩大报销范围。新农合住院补助封顶线提高到15万元。实施残疾人康复等项目，使中重度残疾人普遍受益。实施妇女“两癌”筛查和救助工程。推进养老服务体系建设，完善老年人体育服务设施，建设市居家养老服务信息平台。实施15个城市社区服务体系项目。全面做好人口工作，稳定低生育水平。

　　（六）努力提升城市品位。

　　加大城市规划、建设和管理力度，打造功能完善、环境优美、宜居宜业的休闲养生城市，努力提高群众的幸福指数。

　　强化城市规划工作。按照建设宜居宜业城市的要求，高标准完成铜川市城乡一体化建设规划、中心城区发展战略规划、城市综合交通规划的编制工作。按照做美做靓城市的要求，搞好城市重点区域景观设计，做好新区空间规划。按照建设“现代耀州”的要求，做好耀州城区改造提升规划。

　　加大城市管理力度。理顺城市管理体制，管理重心下移，夯实区、街道办两级的管理责任。完善长效管理机制，推进城市管理科学化、精细化。强化城市管理执法，集中开展城市规划区私搭乱建专项整治活动。提高市民的城市意识，动员全社会积极参与城市建设和管理。

　　完善城市服务功能。加大新区开发建设力度，完善新区城市雨水收集管网规划，启动重点区域内涝治理工程，实施朝阳路西段、长虹南路辅道建设、九州东道改扩建和华夏大道绿化、景观照明工程。继续抓好北市区片区改造，推进沉陷区滑塌区综合治理，实施山城路改造。加快医源性废弃物、生活和建筑垃圾处理场建设。巩固深化210国道沿线环境综合整治。推进城市集中供热工程。新增天然气用户1万户。

　　加快生态环境建设。认真实施生态立市战略，扎实推进“四城联创”，巩固提升省级园林城市创建成果，完成国家卫生城市、全国绿化模范城市和省级环保模范城市创建年度任务。加大主要街区有碍观瞻破旧建筑拆除改造力度。加大森林资源管理保护力度，认真实施绿化铜川五大工程，完成植树造林15万亩，治理水土流失150平方公里。进一步加大药王山周边水泥粉尘污染治理力度，大力实施电力、水泥行业脱硝工程和机动车辆污染减排工程，万元生产总值二氧化碳排放降低3.5%，确保城市空气质量二级以上天数达到300天以上。认真落实渭河流域水污染防治三年行动方案，抓好市域水污染防治，实施北市区中水回用工程、新耀污水处理厂污泥处置工程，完成鸭口、徐家沟、金华山煤矿污水处理工程建设，确保出市断面水质达到功能区要求。

　　推进城镇化进程。抓好黄堡省级重点示范镇建设，市财政每年安排2000万元，连续三年支持黄堡镇建设。完善镇区水电路气讯和垃圾、污水处理等基础设施，以及幼儿园、学校、医院、商业、文化等基本公共服务设施，按照新型社区标准兴建居民小区，力争使重点示范镇建设一年打基础、两年出形象、三年见成效。实施照金镇片区改造、供水和污水处理等项目，推进照金红色文化名镇建设。加快宜君县城建设。实施新区城中村改造、华原新城建设和耀州古城、锦阳新城建设。推进有条件的农村居民进城落户，优先解决在城镇出生的非城镇人口，完成1.7万农村居民进城落户任务。

　　（七）继续深入推进改革开放。

　　深化重点领域改革。完善国有资本有进有退、合理流动机制，做好国企厂办大集体企业剥离改制和铜变集团重组工作。深化财政体制改革，增强财政保障能力，保工资、保运转、保“科学发展双十行动”。认真落实国家税费改革政策，继续深化国库集中支付制度改革，实现公务卡改革全市预算单位全覆盖，推进“三公”经费和部门预算公开。加大专项资金统筹力度，调整完善重点示范镇财政管理体制。稳妥推进价格改革，抓好煤炭价格调节基金征收工作。加强金融生态环境建设，实施“双推双增”工程，加快企业债券发行工作。推进融资方式创新，加快组建民营投资公司，努力扩大融资规模。鼓励有条件的企业上市融资，并予以奖励。继续推进统筹科技资源改革、集体林权制度配套改革。全面启动县级公立医院改革，做好基层医疗机构药品“三统一”工作。积极推进事业单位分类改革。完善人才培养、引进、使用和奖励机制，为全市经济社会发展提供有力的人才保证。

　　进一步深化文化体制改革。大力实施文化兴市战略，认真贯彻落实市委关于推动文化大发展大繁荣的实施意见，巩固经营性文化单位改制成果，深化文化事业单位改革，完善文化创新的各项政策，提升公共文化服务水平。深入挖掘铜川文化资源，认真实施核心价值引领、宣传舆论导向、文化事业繁荣、文化产业振兴、公共文化共享、宣传文化人才等文化建设六大工程，打造养生文化、红色文化、耀瓷文化等特色文化品牌，加快文化事业和文化产业发展。

　　大力发展非公有制经济。认真落实中省支持中小企业、非公有制经济发展的各项政策，引导金融机构加大对中小企业的信贷支持，大力推进全民创业，激励民营企业家二次创业，推动中小企业、非公有制经济做大规模、做强实力。全年新增私营企业300户以上，小型微型企业和经营户3000户以上，非公有制经济占比达47%以上。

　　加大招商引资力度。坚持把招商引资作为项目带动战略的总抓手，创新招商工作方式和思路，围绕建设休闲养生城市的目标和全市产业发展规划，扎实做好项目策划、包装和推介工作，变招企业为招产业，变招商引资为招商选资。组织好我市赴珠三角、长三角、环渤海、港澳台等地的招商活动，精心做好西洽会、厦洽会、农高会的参会工作。抓住“央企进陕”的机遇，加强与大企业、大集团的合作，提高招商引资的针对性和实效性。各区县每季举办一次专题招商活动，市上每半年组织一次项目观摩会，对招商引资工作进行现场点评。加大考核力度，对招商引资成绩突出的给予重奖。全年招商引资合同利用资金和到位资金较上年增长30%以上。

　　加快外向型经济发展。出台支持外向型经济发展的政策，积极引进和培育出口骨干企业，加大品牌地产品培育，扩大苹果、服装、医药、电源等产品的出口。全年实现外贸进出口总额1000万美元以上。

　　（八）加强和创新社会管理。

　　提高社会管理科学化水平。强化各级政府特别是基层乡镇政府的社会管理和公共服务职能，加强城乡社区建设，规范居民小区管理，充分发挥基层群众自治在社会管理中的作用。认真破解社会管理中的薄弱环节，提高对空巢老人、留守儿童、流动人口的服务水平，加强信息网络管理，搞好非公组织、社会组织的管理。深入推进应急管理工作，加强预案体系、避难场所、自然灾害防御、救援队伍和保障能力建设。深入实施“六五”普法。抓好社会诚信体系建设，强化诚信教育，建设覆盖全社会的诚信系统。加强国防教育，搞好民兵预备役工作，广泛开展双拥共建活动，进一步巩固和发展军政军民团结的大好局面。

　　切实抓好安全生产工作。全面落实中省市加强安全生产的一系列规定和措施，以事故预防为主攻方向，层层落实安全生产目标责任制，严格“一岗双责”、“一票否决”等制度，进一步夯实企业的安全生产主体责任，强化各级政府和部门的安全生产监管责任。扎实推进企业标准化达标建设，打造本质安全型企业。持续深入开展煤矿、道路交通、非煤矿山、危险化学品、民爆物品、烟花爆竹、建筑施工、特种设备、消防等重点领域的安全专项整治，落实黄橙红警示制度和安全生产情况通报制度，提高安全隐患排查治理的效率和质量，有效防范和坚决遏制重特大事故，促进安全生产形势持续稳定好转。强化食品药品监管，开展食品基本品种和药品监督性抽验，杜绝重大食品药品安全事故的发生。严格落实学校、幼儿园安全保障措施，将校车安全纳入各级政府安全监管的重点，营造全社会重视、关爱学生安全的浓厚氛围。

　　全力维护社会和谐稳定。深入开展“平安铜川”创建活动，完善社会治安防控体系，加强维稳反恐应急处突工作，持续开展严打整治和打黑除恶专项斗争，增强群众安全感。加强社会稳定风险评估，以深入开展领导接访为抓手，用群众工作统揽信访工作，积极开展“基层信访能力提高年”、“入百企进千村访万户”及“四无”区县创建活动，完善基层矛盾纠纷排查化解机制，进一步畅通信访渠道，确保实现“一控制、两下降”目标。

　　三、全面加强政府自身建设

　　今年的目标任务艰巨繁重，对政府工作提出了新的更高的要求。我们要坚持依法行政，切实改进作风，优化政府服务，狠抓工作落实，努力提高行政效能和服务水平。

　　加快转变政府职能。不断深化行政管理体制改革，继续精简、规范行政审批事项，切实转变政府管理经济的方式，发挥市场配置资源的作用，加强市场监管、社会管理和公共服务职能。深化政务公开，完善政府新闻发布制度，大力推行电子政务，加强市、区县政府门户网站建设，努力提高政府工作的透明度。坚持从严治政，大力加强公务员教育和管理，增强公务员的责任意识、勤政意识、廉政意识、服务意识和诚信意识。

　　深入推进依法行政。严格依照法定权限和程序行使权力、履行职责，自觉接受市人大及其常委会的依法监督，主动接受市政协的民主监督，认真听取民主党派、工商联、无党派人士和各人民团体的意见。高度重视司法监督、舆论监督和社会公众监督。完善重大事项公众参与、专家咨询论证和政府集体决策相结合的机制，提高决策科学化、民主化水平。严格执行行政执法责任制和执法过错追究制，加强行政复议，依法纠正不当和违法行为，不断提高政府公信力。

　　切实改进工作作风。深入开展以“三进三服务”为主要内容的“为民服务零距离”活动，真心实意解民忧、解民怨、解民困，协调解决企业发展和项目建设中遇到的困难和问题。深化行风评议，扩大民意调查范围，推动机关作风建设。全面落实服务承诺、首问负责和限时办结等制度，完善政府绩效评估制度，强化责任，严格问责，提高政府执行力。完善目标责任考核，狠抓重大项目、重点工作的督促检查，实行市级领导包抓，确保“双十行动”扎实有效推进。

　　不断加强廉政建设。严格落实党风廉政建设责任制，探索建立廉政风险防控机制，加强对行政权力的监督制约。严格执行部门预算、政府采购、领导干部经济责任审计等制度，从严控制各项行政开支，从严控制行政机关办公楼和培训中心等楼堂馆所建设，做到出国出境经费、公务用车购置及运行费、公务接待费零增长。坚持厉行节约，建设节约型机关。筹建市公共资源交易中心。继续开展各类专项治理，规范职务消费行为，加强纠风工作，坚决纠正损害群众利益的不正之风。

　　各位代表，做好今年的工作，责任重大，使命光荣。让我们高举中国特色社会主义伟大旗帜，以科学发展观为指导，在市委的坚强领导下，凝聚各方智慧和力量，进一步解放思想，开拓进取，心无旁骛，真抓实干，以优异成绩迎接党的十八大和省第十二次党代会胜利召开！
